# Delphinidin-3-Glucoside Protects against Oxidized Low-Density Lipoprotein-Induced Mitochondrial Dysfunction in Vascular Endothelial Cells via the Sodium-Dependent Glucose Transporter SGLT1

**DOI:** 10.1371/journal.pone.0068617

**Published:** 2013-07-18

**Authors:** Xin Jin, Long Yi, Ming-liang Chen, Chun-ye Chen, Hui Chang, Ting Zhang, Li Wang, Jun-dong Zhu, Qian-yong Zhang, Man-tian Mi

**Affiliations:** Research Center for Nutrition and Food Safety, Institute of Military Preventive Medicine, Third Military Medical University, Chongqing Key Laboratory of Nutrition and Food Safety, Chongqing Medical Nutrition Research Center, Chongqing, P. R. China; University of Salento, Italy

## Abstract

Delphinidin-3-glucoside (Dp) is a member of a family of bioactive compounds known as anthocyanins that occur naturally in pigmented plants and are known to ameliorate oxidative stress. Previous studies have showed that Dp decreased oxidative stress in vascular endothelial cells, however, the underlying mechanisms remain largely unknown. In the present study, we showed that pretreatment with Dp significantly suppressed oxidized low-density lipoprotein (oxLDL)-induced cell proliferation inhibition and apoptosis in primary human umbilical vein endothelial cells (HUVECs). Also, Dp pretreatment attenuated oxLDL-induced mitochondrial dysfunction via decreased reactive oxygen species (ROS) and superoxide anion generation, thereby repressing mitochondrial membrane potential and closing mitochondrial permeability transition pore. Furthermore, in vitro and in vivo data showed that Dp was transported into endothelial cells in a temperature, concentration, and time-dependent manner via the sodium-dependent glucose transporter (SGLT1). Suppression of SGLT1 by its substrate glucose, its inhibitor phlorizin or SGLT1 siRNA blocked Dp transportation. Repression of SGLT1 significantly inhibited Dp function of ameliorating mitochondrial dysfunction induced by pro-apoptotic factors (Apoptosis-inducing factor, Cytochrome c, Caspase-3 and Bax/Bcl-2 ratio). Taken together, our data indicate that Dp protects VECs via the SGLT1-ROS-mitochodria pathway. This new insight may help to elucidate the molecular mechanisms underlying the vascular protection afforded by Dp, and anthocyanins in general, in the context of prevention of endothelial dysfunction and atherosclerosis.

## Introduction

Endothelial dysfunction is a driving force in the initiation and development of atherosclerosis (AS) [1]. Although AS development appears to be the result of multiple maladaptive pathways, a particularly important risk factor in the pathogenesis of AS is oxidized low-density lipoprotein (oxLDL), which contributes to endothelial dysfunction [2]. Increased production of intracellular reactive oxygen species (ROS) by vascular endothelial cells (VECs) is induced by various stimuli, including inflammatory factors, shear stress, radicals, and in particular, oxLDL. Harmful ROS play a key role in development of endothelial dysfunction [3]. In view of the utilization of most oxygen in the respiratory chain, mitochondria have been presumed as the major source of cellular ROS generation and a portion of electron leakage during oxygen consumption [4], [5]. Thus, augmented formation of mitochondrial ROS appears to be a principal pathway of endothelial dysfunction in AS. On the other hand, mitochondria are very susceptible to ROS attack, as the mitochondrial compartments contain large amounts of unsaturated phospholipids, but have fragile antioxidative defensive systems [6]. In fact, mitochondria are both important sources and targets of ROS [7]. The mitochondrial dysfunction theory postulates that excessive mitochondrial ROS release can contributes to the inflammatory vascular reaction leading to the development of atherosclerotic lesions [8]. As a result, antioxidant supplementation is a plausible strategy to maintain the redox homeostasis by directly quenching excessive ROS, to protect or reinforce the endogenous antioxidative defense system against oxidative stress. Hence, this is the impetus to the ongoing efforts to develop “mitochondriotropic” antioxidants, with the main goal of counteracting these undesirable redox-mediated effects [9]–[11].

**Figure 1:**
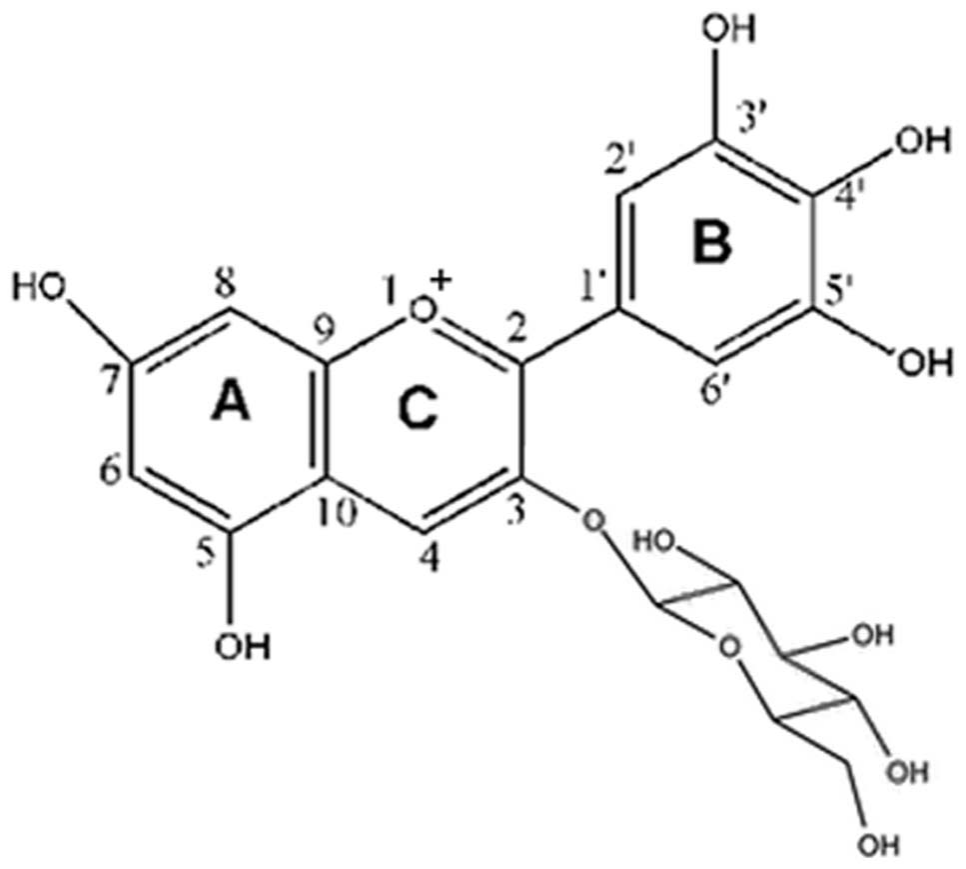
The chemical structure of delphinidin-3-glucoside (Dp).

Anthocyanins (ACNs) belong to family of compounds known as flavonoids, which are part of an even larger group of compounds known as polyphenols [12], [13]. These phenolic compounds are widely found in berries, red grapes, purple sweet potatoes, red cabbages, and many other pigmented foods, plants, and vegetables. There have been over 400 ACNs identified in nature and Epidemiological studies have revealed an inverse association between dietary ACNs intake and reduced occurrence of cardiovascular diseases, such as AS [14], [15]. It is generally considered that these protective activities are related to the antioxidant properties of ACNs which, in turn, are determined by the number and position of hydroxyl groups as substituents and the extent of glycosylation [16]–[19]. Growing experimental data have illustrated that ACNs exhibit favorable antiatherosclerotic effects against endothelial dysfunction by counteracting oxidative stress [20], [21]. Our previous work also demonstrated that among naturally occurring ACNs, delphinidin-3-glucoside (Dp) (Fig. 1), a major ACN present in pigmented fruits and vegetables including pomegranates, berries, dark grapes, egg plant, tomatos, carrots and red onion, possesses potent activity to scavenge free radicals to inhibit endothelial oxidative injury [22]–[24]. Nevertheless, the exact molecular mechanisms of these protective effects have not yet been fully elucidated, and little is known about whether Dp possesses mitochondriotropic antioxidant activities. Importantly, serum levels of unmetabolized ACNs peak in the sub- to low-micromolar range and the peak concentration is reached within the first 30 min to ∼2 h. The antiatherosclerotic effects of dietary ACNs (i.e., the huge increase of plasma antioxidant activity after ingestion of ACN-rich foods) appear to be in striking contrast with their poor bioavailability, unless attributable to yet unidentified accumulation in blood vessels, particularly the vascular endothelium. However, membrane transporters that mediate rapid and specific absorption of ACNs by VECs remain unknown in spite of its obvious and fundamental importance [25]–[27].

Herein, we assessed the role of sodium-dependent glucose cotransporter (SGLT1) in Dp internalization and the inhibition of mitochondrial dysfunction in VECs. The results showed that Dp attenuated oxLDL-induced mitochondrial dysfunction via SGLT1 activity.

## Materials and Methods

### Chemicals

Dulbecco's modified essential medium (DMEM) and fetal bovine serum (FBS) were obtained from HyClone Laboratories (Logan, UT, USA). Phlorizin, D-glucose, dimethylsulfoxide (DMSO), and phosphate-buffered saline (PBS) were purchased from Sigma-Aldrich (St. Louis, MO, USA). Dp with a purity >97% was obtained from Polyphenols Laboratories AS (Sandnes, Norway) and was dissolved in DMSO to a final concentration of 50 mM. Native LDL and oxLDL were prepared as previously described [22], and the extent of oxidation of LDL was evaluated by thiobarbituric acid-reacting substances (TBARS) assay (ZeptoMetrix Co.).

### Ethics Statement

Human umbilical vein endothelial cells (HUVECs) were isolated by collagenase digestion of umbilical veins from fresh cords obtained at normal deliveries, which were from donors with written informed consent. The study protocol was approved by the Ethics Review Committee of The Third Military Medical University. All animal experimental procedures were carried out in strict accordance with the recommendations in the Guide for the Care and Use of Laboratory Animals of the National Institutes of Health and the protocol was approved by Animal Ethics Committee of the Third Military Medical University (Chongqing, China) (approval SYXC-2007-0002).

### Cell culture

HUVECs were cultured in DMEM containing 10% FBS, 3.2 mM glutamine and 100 U/mL penicillin–streptomycin, as reported previously [23]. Experiments were performed after passage 3 with 70–80% confluent cells.

### MTT assay

HUVECs were pretreated for 2 h with different concentrations (0.1, 1, 10, 50, 100, and 200 μM, respectively) of Dp. After removing the supernatant, the cells were exposed to 100 μg/mL of oxLDL for an additional 24 h. Then, cells were incubated with 5 mg/mL of MTT for 4 h, and the blue formazan crystals of viable cells were dissolved in DMSO and then measured spectrophotometrically at 490 nm. The cell viability of the control group was set at 100%, and that of the other groups is shown as the percentage of control cells.

### Flow-cytometric analysis of apoptosis

After the indicated treatments, the treated HUVECs were harvested, washed three times with ice-cold PBS, and assessed for apoptosis using an annexin-V-fluorescein isothiocyanate (FITC) and propidium iodide (PI) double staining kit (Clontech, Mountain View, CA, USA) according to the manufacturer's instructions. Briefly, cells were collected by centrifugation, washed twice with cold PBS, centrifuged at 1000 rpm for 5 min, then gently resuspended in 500 μL of binding buffer. Then, 5 μL of Annexin V-FITC and 5 μL PI solution were added and incubated with the cells in the dark for 15 min. At the end of incubation, cell apoptosis was analyzed on a FACScan flow cytometer (FCM) (Becton Dickinson, Franklin Lakes, NJ, USA).

### Fluorescence spectroscopy and imaging

Mitochondrial dysfunction was assessed by measuring the ROS and superoxide anion (O_2_^·−^) scavenging activity, mitochondrial membrane potential (ΔΨm), and mitochondrial permeability transition pore (mPTP) opening. HUVECs were pretreated for 2 h with different Dp concentrations (1, 10, 50 and 100 μM, respectively), washed, and then exposed to 100 μg/mL of oxLDL for 24 h. The determination of intracellular ROS and O_2_^·−^ production were measured by using the fluorescent probes 2′,7′-dichlorofluorescin diacetate (DCFH-DA) [24] and dihydroethidium (DHE) (Sigma-Aldrich) [28], respectively. The fluorescent, lipophilic, and cationic probe, JC-1 (Beyotime Institute of Biotechnology, Beijing, China), was employed to measure the ΔΨm of HUVECs according to the manufacturer's directions, which have been described previously [29]. The mPTP opening of HUVECs were detected by calcein–cobalt staining using an mPTP assay kit (Genmed Scientifics Inc., Wilmington, DE, USA) according to the manufacturer's directions as described previously [30]. Images were obtained via confocal laser scanning microscope (CLSM; model TCS SP2; Leica Microsystems GmbH, Wetzlar, Germany) and FCM (for JC-1 only), respectively. In different measurements, fluorescences were determined using an Infinite^TM^ M200 Microplate Reader (Tecan Group Ltd., Männedorf, Switzerland). The amount of emitted fluorescence after DCFH-DA and DHE loading were correlated with the quantities of ROS and O_2_^·−^ in the cell and the intracellular fluorescence intensity was expressed as the fold change relative to the control group, respectively. The ΔΨm measured by spectrophotometry assay was calculated as the ratio of red to green fluorescence, and that detected by flow cytometry was expressed as percent cells with decreased ΔΨm (R4 quadrant). The fluorescent signals in the mPTP assay were normalized to total protein content in the corresponding cell extracts and results were presented as normalized relative fluorescence units (NRFU; U/mg of protein). All experiments were repeated at least three times.

### CLSM and HPLC analysis

Identification of Dp in HUVECs was determined by CLSM and HPLC analysis by taking advantage of the autofluorescent property of Dp. Briefly, HUVECs were treated with 10 μM Dp for 2 h. And then, all dishes were submitted to fluorescence spectral analysis using a CLSM (TCS SP2; Leica Microsystems GmbH). The Dp fluorescence spectra was measured in extracts at pH 3.0. Additionally, treated cells were lysed by repeated shock freezing in liquid nitrogen and thawing. Next, the supernatant was dissolved in ethyl-acetate and dried under nitrogen gas at room temperature. Dried extracts were suspended in 600 µL of methanol (pH 3.0) for HPLC analysis. The chromatographic analysis was carried out using an HPLC-UV-diode array detector and Phenomenex Prodigy C18 column (5 μm; 4.6×250 mm; ODS-3 100 Å; Phenomenex, Inc., Torrance, CA, USA). The mobile phase (methanol:water) ratio was 48∶52 and the flow rate was 1 mL/min. Dp was identified by comparing spectral features and retention time of the compound with a pure standard. Quantification was calculated using a calibration curve of pure standard compounds run under the same conditions.

Furthermore, the time, dose, and temperature dependencies of Dp uptake by HUVECs were measured via HPLC. Briefly, cells were pre-incubated with 10 μM Dp for different time intervals (10 min, 30 min, 1 h, 2 h, 4 h, and 24 h, respectively) at 37 and 4°C, respectively. Afterward, the cells were washed with cold PBS, resuspended in ethanol, and then transferred to a quartz cell to measure cellular density and viability using a CASY TT Cell Counter and Analyzer (Roche Diagnostics, Mannheim, Germany) according to the manufacturer's instructions. Next, the HUVECs were treated with various Dp concentrations (1, 10, 20, 50, and 100 μM, respectively) for 2 h at 37 or 4°C, then Dp uptake was measured by HPLC. The intracellular Dp content per 10^6^ cells was calculated and all experiments were repeated at least three times.

### Animal study

**Figure 2:**
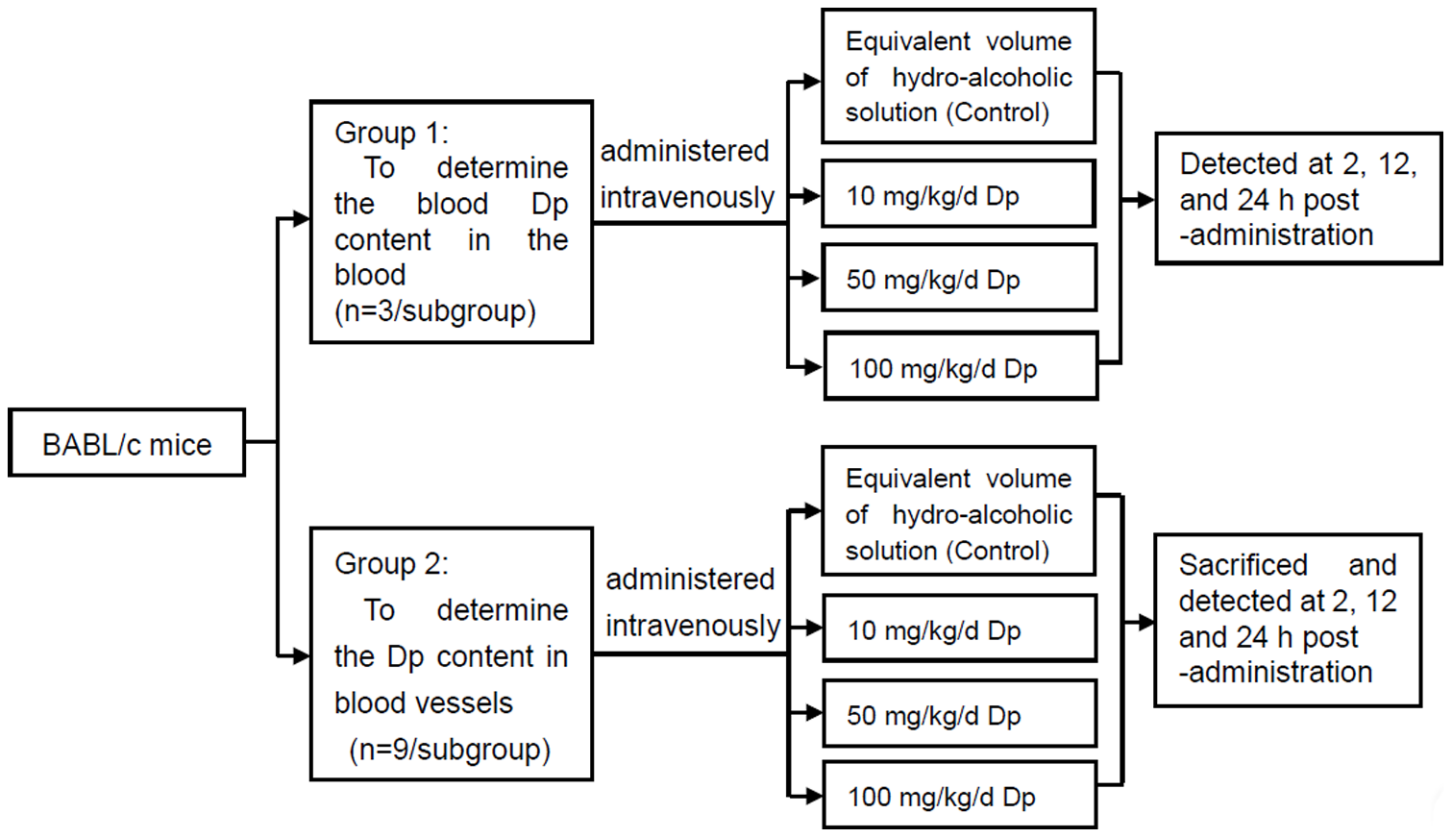
Experimental design. The schematic depicts the overall design of the animal experiment to evaluate the Dp content in the blood and blood vessels of BABL/c mice.

Forty-eight female adult BABL/c mice (body weight, 15–18 g) were housed in cages under controlled conditions of a 12-h light:dark cycle at a temperature of 22±3°C and a relative humidity of 40–70%. The mice were supplied with standard laboratory chow and water ad libitum. No traces of ACNs were detected in the commercial diet, as revealed using the method described by Juan et al. [31]. Mice were fasted overnight and then randomly divided into two groups to detect of the Dp content in the blood and blood vessels (Fig. 2). Mice were intravenously administered a hydro-alcoholic Dp solution at different concentrations (10, 50, and 100 mg/kg/day, respectively) or the same volume of the hydro-alcoholic solution without Dp (as a control). Then, the Dp content was evaluated by HPLC at different time intervals (2, 12, and 24 h, respectively) after the blood and thoracic aorta samples were treated according to the procedures proposed by Biasutto et al. [32] and Bertelli et al. [33]. The thoracic aortas of the mice in group 2 were separated, cut transversely into 5-mm sections using a cryostat (Leica Microsystems GmbH) and then mounted on glass slides. The frozen sections were stained with the fluorescent probe PI (Vector Laboratories, Inc., Burlingame, CA, USA) to stain the nuclei. Each frozen section was evaluated for a fluorescent signal for Dp in blood vessels using a CLSM (TCS SP2; Leica Microsystems GmbH).

### Immunofluorescence analysis

Confluent HUVECs were grown on glass coverslips and then harvested. The thoracic aortas of BABL/c mice were isolated and prepared for frozen sections. All samples were fixed with 4% (v/v) paraformaldehyde for 20 min and then permeabilized with 0.1% Triton X-100 for 10 min at room temperature. The coverslips and frozen sections were blocked with PBS supplemented with 0.1% (w/v) BSA and 0.1% (w/v) Tween 20, and then incubated with anti-SGLT1 antibody (1∶500 dilution; Sigma-Aldrich) overnight at at 4°C. After washing with PBS, the slides and frozen sections were covered for 1 h at room temperature with a FITC-conjugated secondary antibody (1∶1,000 dilution; Molecular Probes, Eugene, OR, USA). After washing, the cell nuclei were counterstained with 4′,6′-diamidino-2-phenylindole for 5 min. Images of the stained cells were obtained using CLSM (TCS SP2; Leica Microsystems GmbH).

### Role of SGLT1 in cellular uptake studies

The role of SGLT1 in Dp uptake by HUVECs was assessed using Na^+^, D-glucose, and phlorizin, respectively. Briefly, HUVECs were cultured in 6-well plates and then exposed to 10 μM Dp with Na^+^-supplemented DMEM, Na^+^-free DMEM, and D-glucose at different concentrations (10, 30, and 100 mM, respectively), and phlorizin (0.25 and 0.5 mM, respectively) for 2 h at 37°C. The control cells were treated with 0.1% DMSO. The intracellular content of Dp per 10^6^ cells in the treated groups was measured via HPLC. Furthermore, SGLT1 was knocked-down by transfecting HUVECs with 40 nM of small interfering RNA (siRNA) (Santa Cruz Biotechnology, Inc., Santa Cruz, CA, USA) according to the manufacturer's protocol. The SGLT1 mRNA expression was determined by real-time reverse-transcription polymerase chain reaction (qRT-PCR). After a 4-h transfection period, the cells were switched to complete DMEM and incubated for 24 h. Next, the transfected cells were exposed to different doses (1, 10, 50, 100 and 200 µM, respectively) of Dp for 1 h at 37°C. Thereafter, the intracellular Dp content per 10^6^ cells was measured by HPLC.

### Western blotting

HUVECs were seeded in 6-well plates at an initial concentration of 1×10^6^ cells/well. Cells were pretreated for 2 h with different Dp concentrations (1, 10, and 100 μM) and 10 μM Dp combined with 40 nM SGLT1 siRNA or 0.25 mM phlorizin as well as the corresponding amount of solvent, respectively. After the supernatant was removed, the cells were exposed to 100 µg/mL of oxLDL for 24 h. Cytosolic fraction proteins were prepared using Cytoplasmic Extraction Reagents (Thermo Fisher Scientific, Waltham, MA, USA) according to the manufacturer's instructions. Equal amounts of protein (30 µg) in the presence of 5% β-mercaptoethanol were electrophoresed on 8% (β-actin and apoptosis-inducing factor (AIF)) or 12% (Bcl-2, Bax, cytochrome c (Cyt c), and Caspase-3) sodium dodecyl sulfate polyacrylamide gel. Proteins were transferred to Immobilon-P membranes (Millipore, Billerica, MA, USA) by electroblotting at 30 V overnight at 4°C. Membranes were blocked for 1 h with 5% nonfat milk in PBS plus Tween 20 (PBST), and incubated with specific primary antibodies against Cyt c, AIF, Bax, Bcl-2, Caspase-3, and β-Actin (Santa Cruz Biotechnology, Inc.) at 4°C overnight. The membrane was washed with PBST buffer and incubated with secondary antibodies (Beyotime Institute of Biotechnology) conjugated with horseradish peroxidase. The specific proteins were detected using enhanced chemiluminescence kits (Amersham, Buckinghamshire, UK) according to the manufacturer's instructions. Densitometric evaluation of the photographic films was performed using Integrated Performance Primitives 6.0 software (Intel Corp., Santa Clara, CA, USA). The experiment was repeated three times and the protein expression was presented as the fold change relative to the control group.

### Statistical analysis

Results are expressed as the mean ± standard error of the mean (SEM). Raw data was analyzed with SPSS 13.0 software and each experiment was carried out at least three times. Statistical analysis for multiple group comparisons was performed by one-way analysis of variance, followed by post-hoc least significant difference tests. A two-sided *P*-value<0.05 was considered statistically significant.

## Results

### Dp attenuated oxLDL-induced cell proliferation inhibition and apoptosis in HUVECs

**Figure 3:**
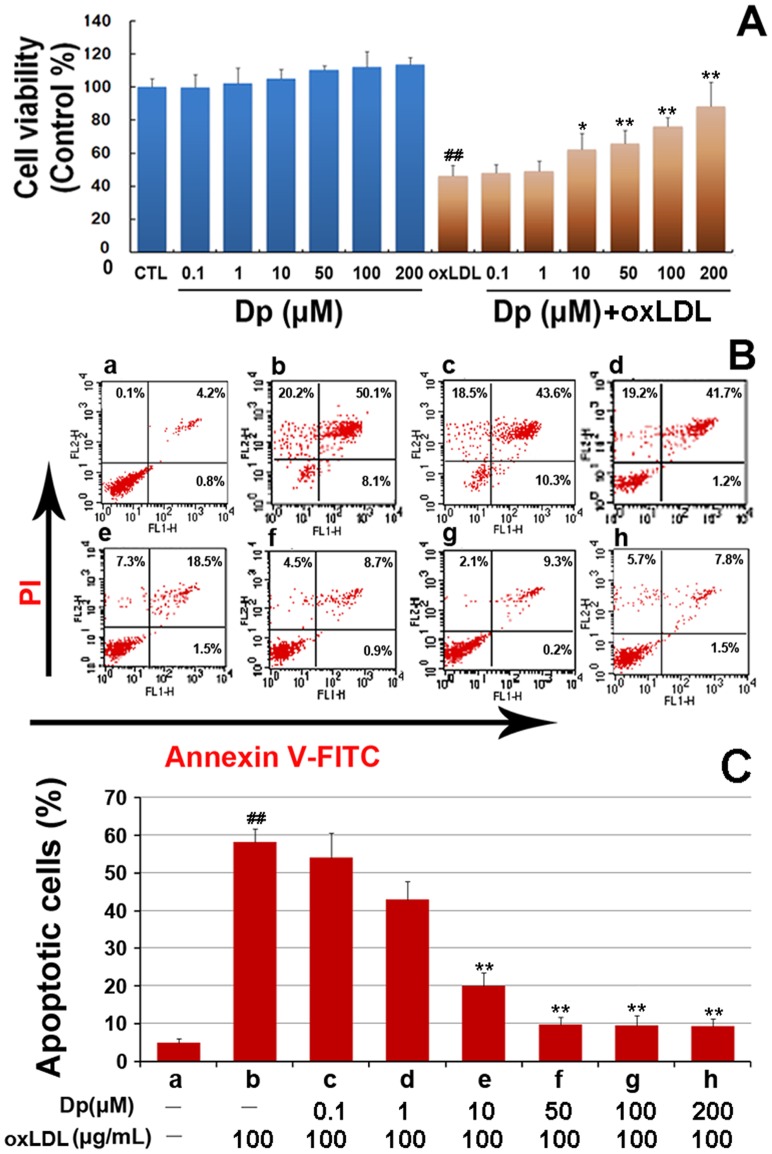
Dp attenuates oxLDL-induced injury in HUVECs. Confluent HUVECs were pretreated for 2 h with various Dp doses (0.1, 1, 10, 50, 100, and 200 μM, respectively). After the supernatant was removed, the cells were exposed to 100μg/mL of oxLDL for 12 h. (A) Cell viability was determined by using MTT assay and data are expressed as the percentage of the control (CTL). There was a significant deterioration in the cell viability parameters after oxLDL treatment compared to CTL (^##^ *P<*0.01) and Dp pretreatment dose-dependently protected against the oxLDL-induced alterations. High Dp concentration ameliorated cell viability (^**^ *P<*0.05). (B) Representative images of flow cytometric analysis by annexin-V-FITC/PI dual staining. The right bottom quadrant represents the Annexin-V-stained cells (early-phase apoptotic cells) and the top right quadrant represents PI- and Annexin V-stained cells (late-phase apoptotic/necrotic cells). (C) Apoptotic cells represent the percentage of Annexin V single positive and Annexin V/PI double positive cells (a-h). All results are presented as mean ± SEM. for at least three independent experiments. ^##^ *P*<0.01,vs. the control group; ^*^ *P*<0.05,^**^ *P*<0.01 vs. oxLDL-treated group.

To investigate the protective effects of Dp on oxLDL-induced HUVECs injury, cells were pretreated for 2 h with a series of Dp concentrations (0.1, 1, 10, 50, 100, and 200 μM, respectively). As shown in Fig.3A, there was no significant differences in cell viability in Dp-treated HUVECs compared to the control group (*P*>0.05), while Dp pretreatment with 10, 50, 100, and 200 μM significantly attenuated the decrease in cell viability caused by oxLDL treatment (*P*<0.05). Moreover, we investigated the effect of Dp on oxLDL-induced apoptosis in HUVECs using an Annexin V-FITC apoptosis detection kit (Figs. 3B and C). More apoptotic cells were observed in oxLDL-treated cultures, yet pretreatment with Dp at 10, 50, 100, and 200 μM significantly reduced the amount of apoptotic cells in comparison to the oxLDL-treated group (*P*<0.05).

### Dp inhibited oxLDL-induced mitochondrial dysfunction in HUVECs

**Figure 4:**
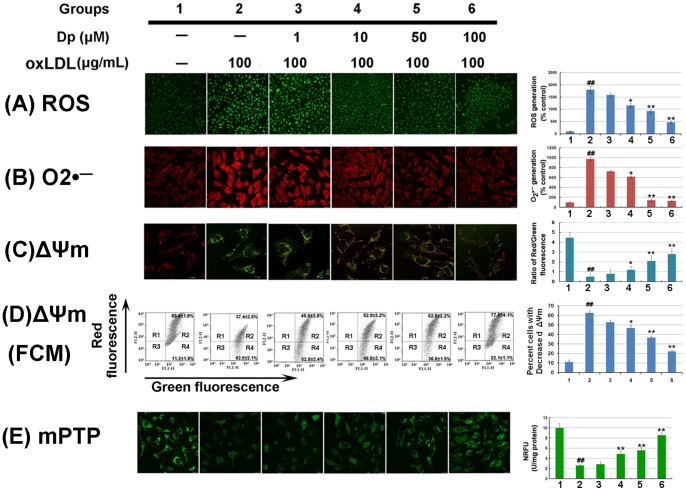
Effects of Dp on mitochondrial dysfunction in oxLDL-induced HUVECs. HUVECs were pretreated with various Dp concentrations (1, 10, 50 and 100 μM, respectively) for 2 h, and then exposed to 100 μg/mL of oxLDL for another 24 h. (A) Intracellular ROS levels were estimated using the probe DCFH-DA. Fluorescence was read at 485 nm for excitation and 520 nm for emission. (B) Intracellular O_2_^·−^ levels were estimated using the probe DHE. Fluorescence was read at 355 nm for excitation and 420 nm for emission. (C) Determination of ΔΨm was carried out using CLSM and fluorescences were determined by spectrophotometry assay. Red fluorescence is emitted by JC-1 aggregates in healthy mitochondria with polarized inner mitochondrial membranes, while green fluorescence was emitted by cytosolic JC-1 monomers and indicates ΔΨm dissipation. Merged images indicated the co-localization of JC-1 aggregates and monomers. The ΔΨm in each group was calculated as the ratio of red to green fluorescence. (D) ΔΨm was detected by flow cytometry assay. The designated R2 and R3 regions represent cell populations that exhibit high (R2) or low (R4) red-to-green fluorescence ratio, consistent with high and low Δψm, respectively. Results shown are one representative of three separate experiments and the proportion of cells in R3 regions were quantified and expressed as percent cells with decreased ΔΨm. (E) The mPTP opening was assayed using the calcein–cobalt quenching method. Different HUVEC groups were used to measure the normalized relative fluorescence units (NRFU) of calcein. Fluorescences were determined using an Infinite^TM^ M200 Microplate Reader (Tecan Group Ltd., Männedorf, Switzerland). All results are presented as means ± SEM. and images were representative of at least three independent experiments. ^##^P<0.05, versus the control group; ^*^P<0.05, ^**^P<0.01 versus oxLDL-treated group.

To investigate the effects of Dp on mitochondrial function, indicators of mitochondrial activity, such as ROS and O_2_^·−^ levels, membrane potential (ΔΨm), and mPTP opening, were evaluated. As illustrated in the histograms of in Figs. 4A–E, oxLDL exposure significantly increased ROS and O_2_^·−^ production in compared to that of the control group (*P*<0.01; Figs. 4A2 and B2). However, the increased amount of ROS and O_2_^·−^ in the oxLDL group was reduced by Dp pretreatment (*P*<0.05; Figs. 4A3–6 and B3–6). Furthermore, we estimated ΔΨm using the JC-1 probe to detect both polarized (red) and depolarized (green) mitochondria and the calcein–cobalt method was employed to monitor distribution of emitted calcein fluorescence (green) reflecting intact mPTP. When cells were exposed to oxLDL, the ΔΨm was depolarized, as shown by a significant increase in green fluorescence (*P*<0.01; Figs. 4C2). However, pretreatment with Dp notably reduced the change in ΔΨm, as indicated by repression of green fluorescence and restoration of red fluorescence (*P*<0.05; Figs. 4C3–6). Quantitative analysis from flow cytometry supported these findings. As shown in Fig. 4D, Exposure to oxLDL resulted in an increase in the percent cells with decreased ΔΨm (*P*<0.01; Fig. 4D2). However, Dp pretreatment abrogated the insult induced by oxLDL (*P*<0.05; Figs. 4D3–6). Additionally, there was a significant decrease in mitochondrial fluorescence in oxLDL-treated HUVECs with the calcein–cobalt loading compared to the control group (*P*<0.01; Fig. 4E2). However, the Dp-pretreated groups demonstrated significantly higher NRFU levels compared to the oxLDL group (*P*<0.05; Figs. 4E3–6). These findings provided evidence that Dp protected against oxLDL-induced mitochondrial dysfunction in HUVECs.

### Dp was incorporated into VECs

**Figure 5:**
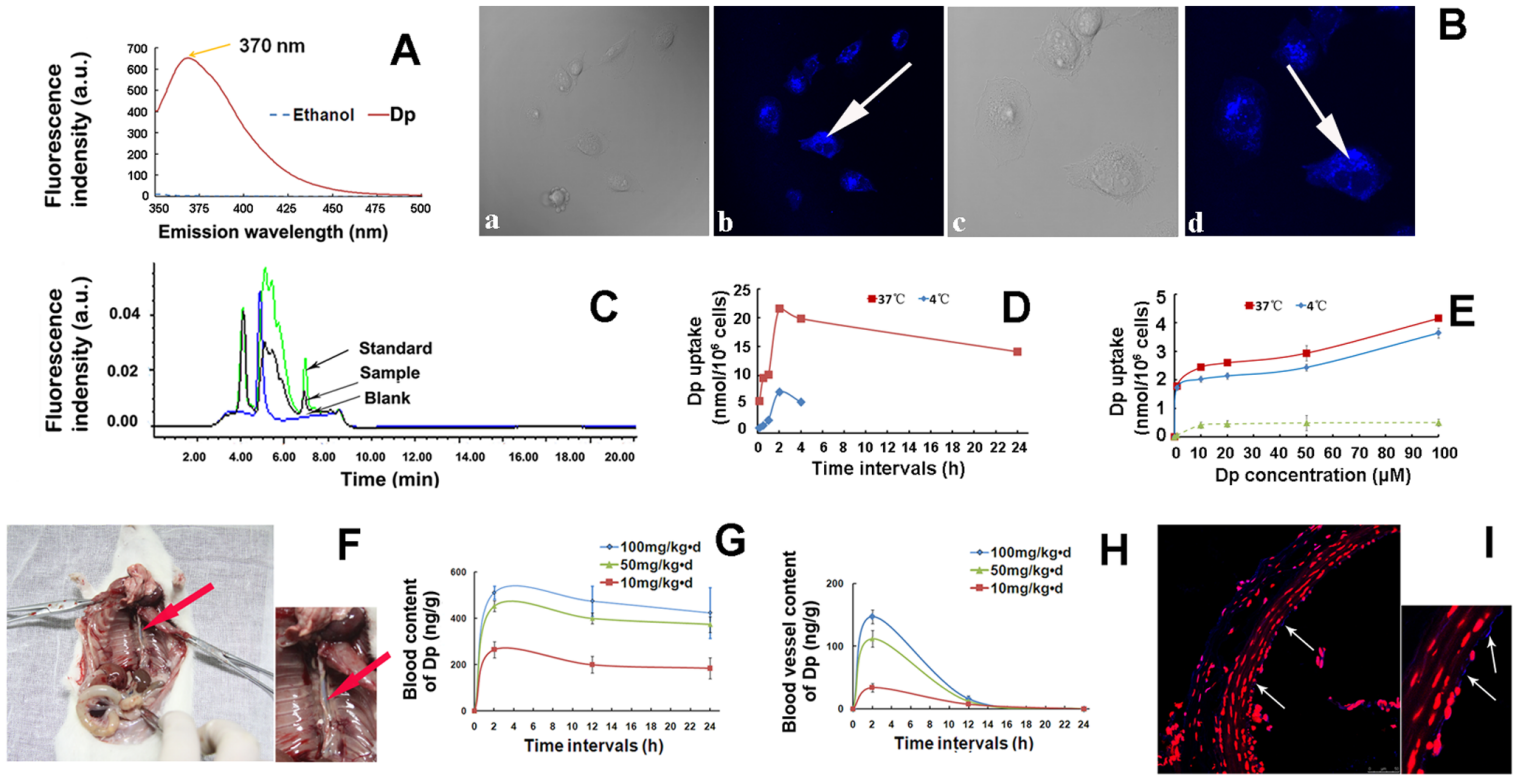
Determination of Dp uptake by VECs. (A) The autofluorescent emission spectra of Dp and ethanol (baseline). The peak emission wavelength was recorded at approximately 370 nm. (B) HUVECs were treated with 10 μM Dp for 2 h and the subcellular Dp distribution was measured using a CLSM (b, ×400 and d, ×1000 magnification) and with a contrast phase microscope (a, ×400 and c,×1000 magnification). (C) Evaluation of intracellular Dp content in HUVECs via HPLC. After incubation, cells were lysed and the supernatant was collected for HPLC analysis. The blank control cells was treated with 0.1% DMSO. The excitation spectra of the fluorescence for λem  = 370 nm is presented in arbitrary units (a.u.). (D-E) Time, dos-, and temperature-dependent uptake of Dp in HUVECs was measured via HPLC. (D) HUVECs were treated with 10 μM Dp at 37 (▪) and 4°C (⧫) for the indicated times (10 min, 30 min, 1 h, 2 h, 4 h, and 24 h, respectively). (E) HUVECs were treated with different Dp concentrations (1, 10, 20, 50, and 100 μM, respectively) for 2 h at 37°C (▪) or 4°C (⧫), respectively. The difference between incorporated Dp by HUVECs at 37°C and that at 4°C represents the actively transported amount (▴). BABL/c mice were intravenously administrated different Dp doses or an equivalent volume of hydro-alcoholic solution. (F) Representative image showed the isolated thoracic aorta (red arrow). The Dp content in the blood (G) and isolated thoracic aorta (H) were measured by HPLC. The isolated thoracic aortas were separated, frozen sectioned, and then stained with PI to visualize the nuclei. (I) Representative fluorescent signals (white arrows) of Dp in blood vessels were observed by by CLSM (TCS SP2, Leica Microsystems GmbH). Values are presented as means ± SEM, n = 3.

To further investigate whether Dp was taken-up by the HUVECs and blood vessels, the autofluorescence property of Dp were identified at an excitation wavelength of 315 nm and a peak emission wavelength recorded at approximately 370 nm (Fig. 5A). After Dp pretreatement for 2 h, some molecules crossed the cell membrane as revealed by cytoplasmic staining (Figs. 5B-b and d), as the blue granular fluorescence was distributed throughout the cytoplasm, with only a weak fluorescence scattered in the nucleus. Additionally, HPLC was used to identify the incorporation of Dp in HUVECs. As shown in Fig. 5C, Dp was detected in the cell lysate, thus providing a direct clue that unmetabolized Dp was internalized by the HUVECs. The intracellular Dp content was 0.006 µg/mL and 0.034 µg/mL following Dp pretreatment at 10 and 100 µM, respectively. Moreover, to determine whether Dp was internalized by the HUVECs by active transport, the time, dose, and temperature dependencies of Dp internalization was assessed. Our results showed that Dp uptake by HUVECs increased rapidly during the first hour and reached a maximum value after approximately 2 h (Fig.5D). A relatively steady-state was observed over a 20-h period after the initial phase at 37°C and the results indicated that Dp uptake occurred in a time-dependent manner and increased with the treated concentrations without saturation at 37 and 4°C (Fig. 5E), suggesting dose-dependency. However, Dp uptake at 4°C was significantly less than that at 37°C using the same dose. Together, these results indicated that Dp uptake at 37°C likely involved two processes, passive diffusion and active transport. As expected, when the amount of incorporated Dp measured at 37°C was corrected from the passive amount measured at 4°C, the obtained curve, which appeared to be obviously saturated, corresponded to a carrier-mediated transport process with a saturation concentration of approximately 10 μM. Furthermore, the Dp content in blood and blood vessels was measured after the mice were intravenously administrated various Dp doses. There was a sustained retention of Dp in the blood vessels compared to that in the blood (Figs.4F, G, and H) and Dp was also apparently present in the vascular endothelium of the isolated thoracic aortas (Fig. 5I).

### Dp uptake was mediated by SGLT1

**Figure 6:**
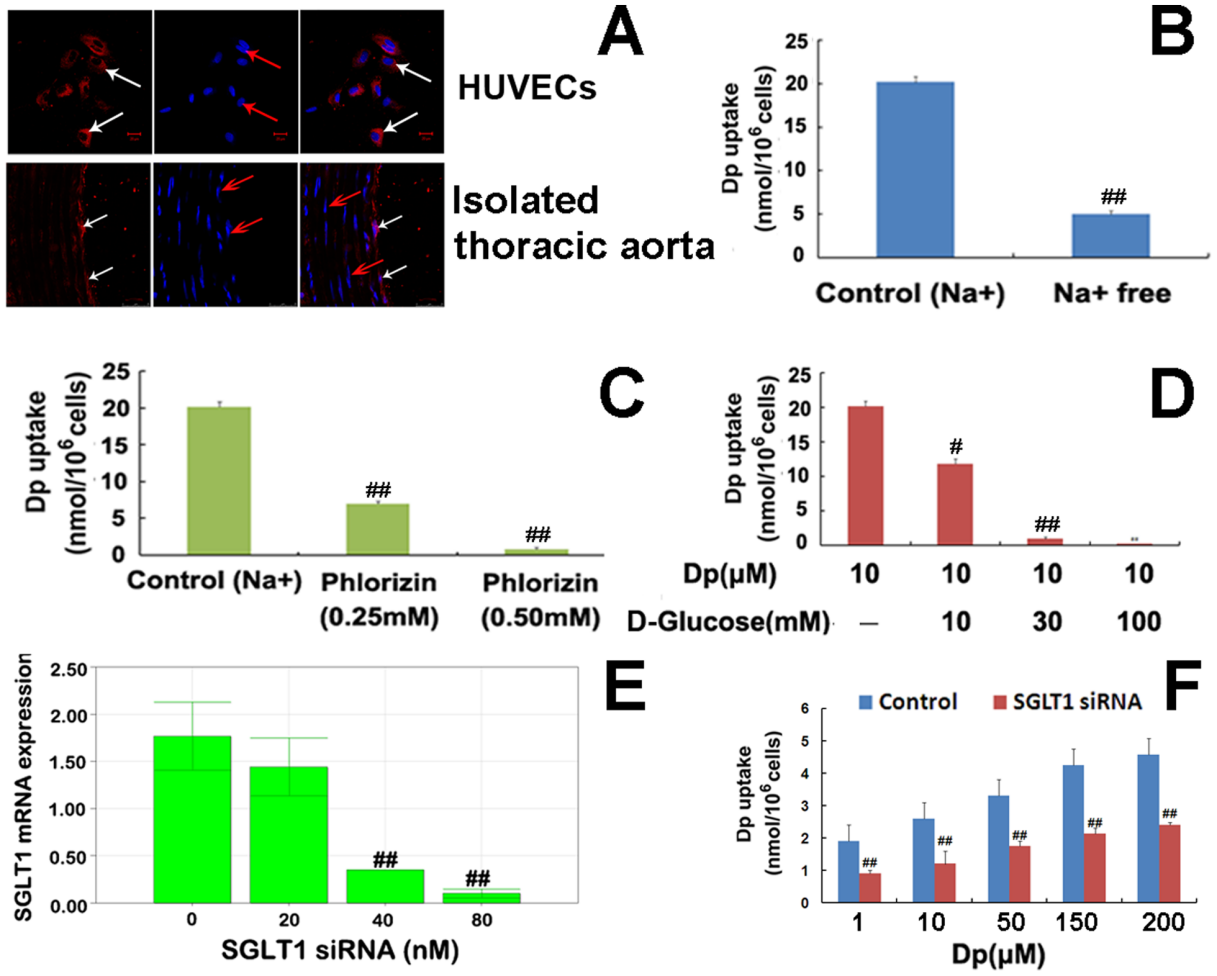
The role of SGLT1 in Dp uptake by HUVECs. (A) Representative images showed SGLT1 expression on HUVECs and the isolated thoracic aorta of BABL/c mice by immuofluorescence assay. Red fluorescence is emitted by SGLT1 immunocomplexes (white arrows) and blue fluorescence is emitted by the nuclei stained with 4′,6′-diamidino-2-phenylindole (red arrows). (B-D) HUVECs were exposed to 10 μM Dp for 2 h at 37°C in the presence of normal sodium buffer (the control), sodium-free buffer, and culture medium supplemented with phlorizin or D-glucose, respectively. After 2 h of incubation, cells were washed and the intracellular Dp content was measured via HPLC. The effects of sodium (B), phlorizin (C), and D-glucose (D) on Dp uptake were detected, respectively. (E) SGLT1 was knocked-down by transfecting HUVECs with different doses of *SGLT1* siRNA and the SGLT1 mRNA expression was determined by qRT-PCR. (F) The transfected cells were exposed to Dp (1, 10, 50, 100 and 200 µM, respectively) for 1 h at 37°C. Thereafter, the intracellular cntent of Dp per 10^6^ cells was measured via HPLC. Values are presented as means ± SEM. (n = 3);^ ##^ *P*<0.01 vs. the control group.

The role of SGLT1 in Dp uptake was assessed. As shown in Fig. 6A, SGLT1 was noticeably expressed in the HUVECs and vascular endothelium of the isolated thoracic aorta. Further, the effects of sodium (Na^+^, a SGLT1 co-transporter), D-glucose (an SGLT1 substrate), and phlorizin (an SGLT1 inhibitor) on Dp internalization by HUVECs were analyzed. As expected, Dp uptake by HUVECs in Na^+^-containing medium was dramatically higher than that in Na^+^-free medium (*P*<0.01; Fig. 6B). However, when pretreated with phlorizin (Fig.6C) and D-glucose (Fig. 6D), Dp uptake by HUVECs was significantly decreased (*P*<0.01). Dp uptake by HUVECs in the presence of 0.50 mM phlorizin resulted in a reduction of approximately 96%, (0.77±0.27 vs. 20.18±0.66 nmol per 10^6^ cells for phlorizin and control groups, respectively; *P*<0.01), whereas a reduction of >95% occurred following treatment with 3 M of D-glucose (0.94±0.28 nmol per 10^6^ cells; *P*<0.01). Furthermore, there was a significant reduction in Dp uptake by *SGLT*1 mRNA suppression (*P*<0.01; Figs. 6E and F). Together, these results support the hypothesis that the carrier-mediated process of Dp uptake was SGLT1-dependent in HUVECs.

### SGLT1 activity is essential for Dp-mediated inhibition of mitochondrial dysfunction

**Figure 7:**
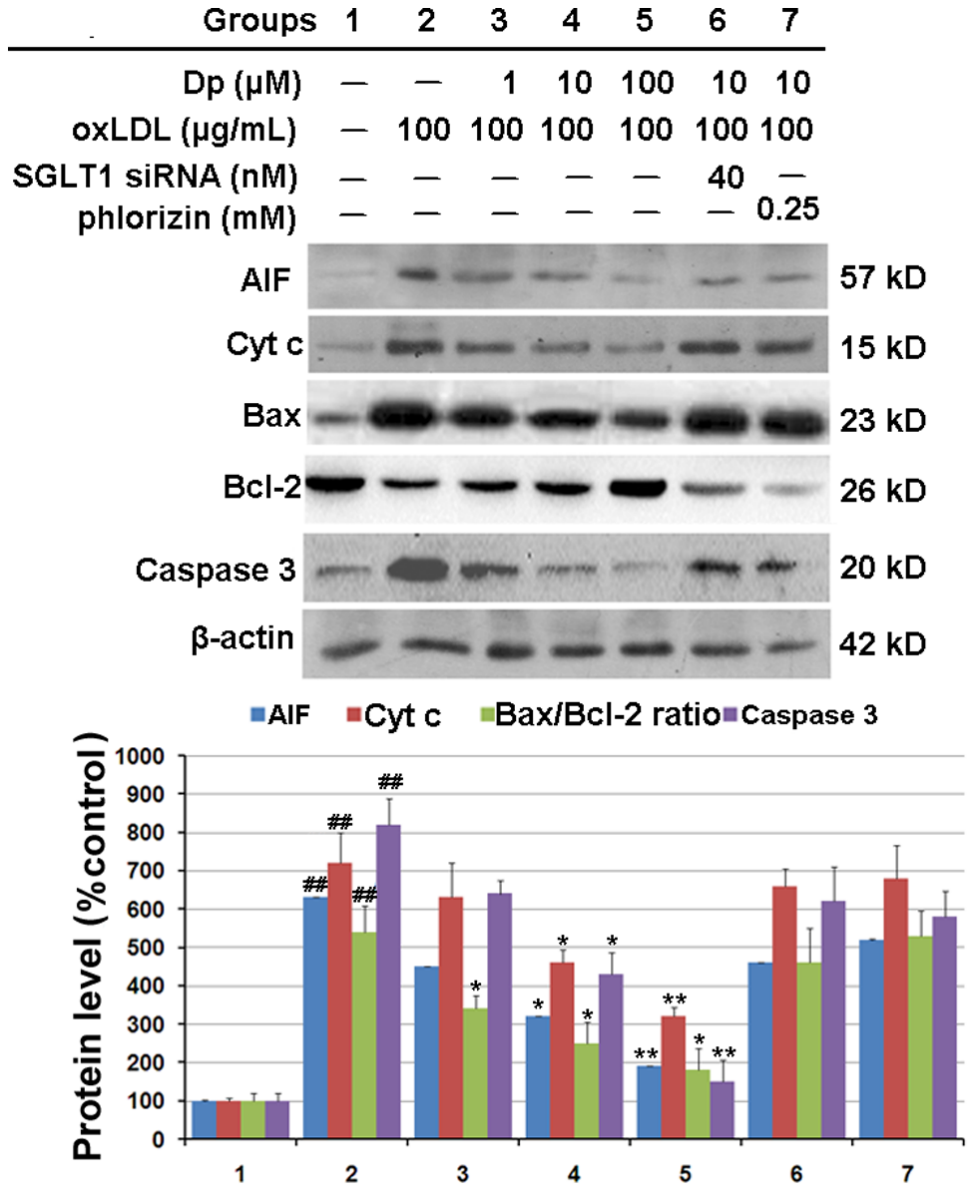
SGLT1 activity is essential for the inhibitive effects of Dp on mitochondrial dysfunction in HUVECs. After the indicated treatements, the cells were harvested and lysed to detect protein levels of AIF, Cyt c, Bcl-2, Bax, Caspase-3, and β-Actin by western blot analysis. The blots are representative of three independent experiments and values are presented as means ± SEM. (n = 3); ^##^ *P*<0.01 vs. the control group; ^*^ *P*<0.05, ^**^ *P*<0.01 vs. the oxLDL-treated group.

Mitochondrial dysfunction can initiate apoptosis by releasing pro-apoptotic factors, including AIF, Cyt c, Caspase-3, and Bax, from the mitochondrial intermembrane space into the cytoplasm. Accordingly, changes in cytosolic AIF, and Cyt C content and a ratio of Bax to Bcl-2 were measured using western blot assay. As shown in Fig. 7, oxLDL treatment increased cytosolic Cyt c, AIF and Caspase-3 content as well as the Bax/Bcl-2 ratio, indicating the release of these apoptotic factors from the mitochondrial matrix and their transport into the cytosol, which was markedly inhibited by Dp pretreatment. However, the inhibition of mitochondria-mediated apoptotic factors expression was attenuated following *SGLT*1 mRNA suppression and by using an SGLT1 inhibitor, suggesting that Dp reduced oxLDL-induced mitochondrial dysfunction through an SGLT1-dependent pathway.

## Discussion

**Figure 8:**
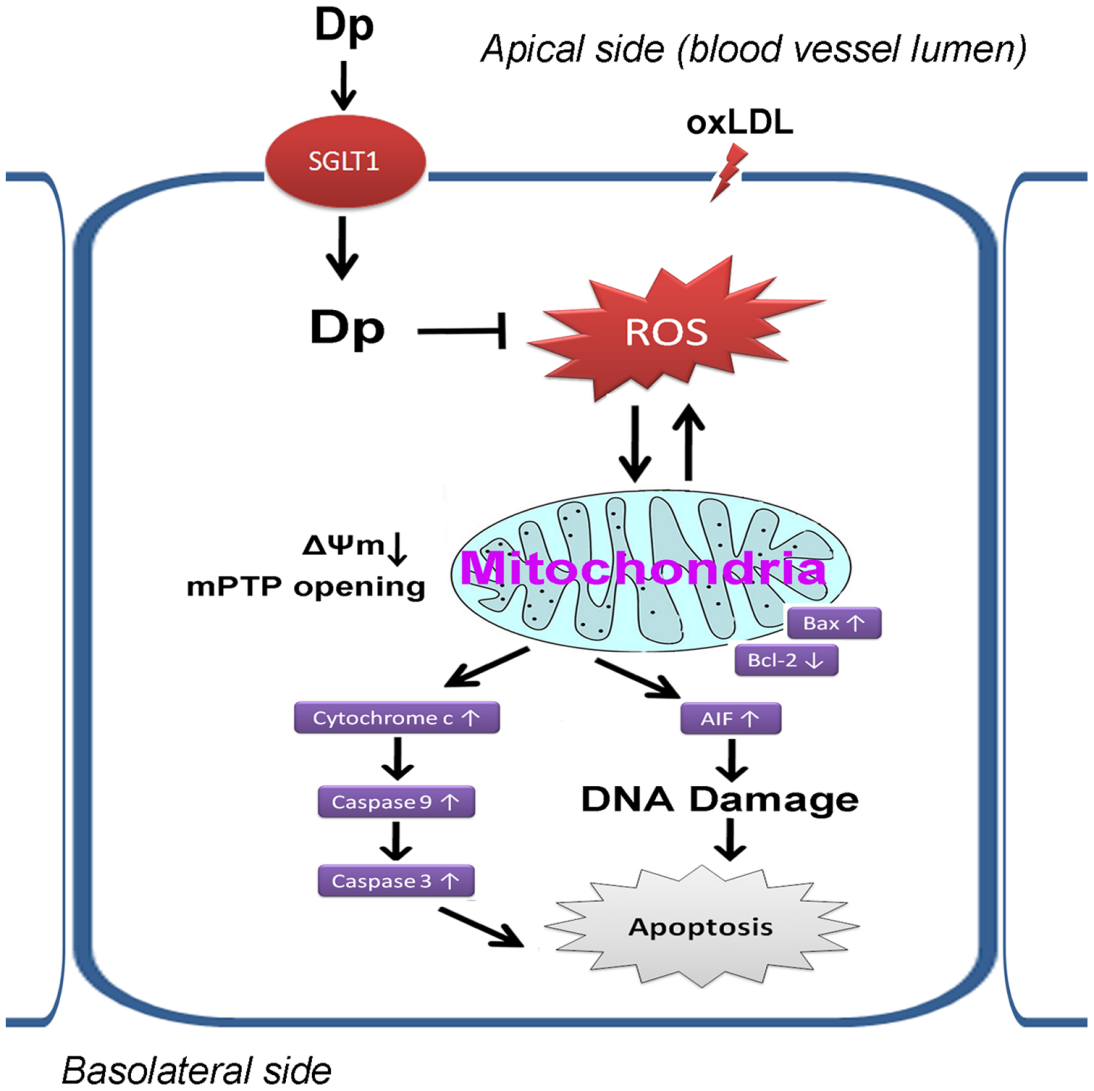
Proposed pathways for Dp uptake by VECs and its inhibitive effects on mitochondrial dysfunction.

The main findings of our study were: (i) oxLDL-induced endothelial dysfunction in primary HUVECs was efficiently prevented by Dp pretreatment through counteracting the mitochondrial apoptotic pathway; (ii) Dp was apparently taken-up by HUVECs and persistently preserved in blood vessels; and (iii) Dp uptake by VECs and its inhibitive effects on mitochondrial dysfunction were dependent on SGLT1 transport activity (Fig. 8). To our knowledge, this is the first report to elucidate the protective action of Dp against mitochondrial dysfunction in HUVECs and to investigate the involvement of SGLT1. Importantly, this study also showed that the biological response of the cells or isolated tissues were secondary to the entry of Dp, and ACNs in general, into the vascular endothelium, and therefore, arose from the interactions between ACNs and intracellular targets.

ACNs are plant pigments that are widely found in many berries, dark grapes, cabbages, and other pigmented foods, plants, and vegetables. Chemically, ACNs belong to the phenolic compounds collectively named flavonoids and have molecular structures based on the 2-phenylbenzopyrylium (or flavylium) cation. ACNs accumulate to high concentrations in some foods (i.e., berries and fruits) and their consumption ranging up to several 100 mg/serving can be achieved. Depending on the nutritional habits, the daily intake of ACNs in humans has been estimated to be up to 200 mg/day [34]. And ACNs have been found to be absorbed unmodified from the diet [35], [36]. It has been proposed that moderate consumption of ACN-rich foods such as red wine or bilberry extract is associated with a lower risk of cardiovascular disease [37]–[41], which is believed to be related to their antioxidant properties [42], [43]. Vascular endothelial dysfunction, mainly induced by oxLDL, is commonly considered to have a pivotal role in AS both in the early stages of lesion formation and later during disease development by inducing atherosclerotic plaque instability [44]. A therapeutically relevant mechanism by which ACNs exert an antiatherosclerotic tendency is the protection of VECs against oxLDL-induced endothelial dysfunction [45]. Furthermore, it is well known that mitochondria are key components to VECs function [46] and that mitochondrial dysfunction is the primary cause of endothelial oxidative injury [47]. However, whether Dp could inhibit oxLDL-induced mitochondrial dysfunction in VECs has not yet been determined. In the present study, the MTT assay showed that Dp pretreatment effectively suppressed oxLDL-induced reduction in cell viability and greatly inhibited apoptosis. Moreover, we confirmed that the anti-apoptotic action of Dp was mediated by suppression of the radical-dependent mPTP opening and ΔΨm loss to block the mitochondrial apoptotic pathway and to further prevent endothelial dysfunction. In the previous work by Xie, et al. [48], it was found that oxLDL induced oxidative stress and apoptosis in endothelial cells, which was associated with the activation of NADPH oxidase, the impairment of mitochondrial respiration chain enzymes, and the disorder of key regulators for apoptosis. However, Dp neutralized the harmful effects of oxLDL on oxidative stress, mitochondrial dysfunction, and apoptosis in cultured vascular EC. And our results were in agreement with the previous findings. O_2_^·−^ formation occurs spontaneously, especially in the electron-rich aerobic environment of the inner mitochondrial membrane within the respiratory chain, but increased ROS production results in oxidative damage to mitochondrial proteins and DNA and can also cause peroxidation of unsaturated fatty acids in the membrane phospholipids, which advances the mitochondrial apoptotic pathway. Consequently, there is considerable interest in the selective blockage of mitochondrial dysfunction to determine the role of mitochondria in redox signaling and to develop appropriate therapies. Towards these goals, several mitochondria-targeted antioxidants that selectively block mitochondrial oxidative damage have been developed [49]. These compounds, such as MitoQ, consist of antioxidants covalently linked to triphenylphosphonium, a lipophilic cation that accumulates several hundred-fold within the mitochondria, which is driven by the large membrane potential (negative inside). Mitochondria-targeted antioxidants have potential as therapeutic agents because they are taken up into the mitochondria in vivo following oral administration [50] and they have also been used to probe the role of mitochondrial ROS production in redox signaling and metabolic regulation [51]. Based on our results herein, the anti-oxidative potential of Dp should be recognized for its ability to preserve the activity of endogenous free radical scavengers that lead to mitochondriotropic antioxidant activity in VECs.

Despite the known *in vitro* therapeutic properties of ACNs and their widespread presence in the diet, many studies to date have indicated that ACNs, including Dp, are rapidly absorbed and eliminated and that their absorption has poor efficiency compared to other polyphenols and flavonoids [52], [53]. Thus, some open questions are, do ACNs enter into VECs and if so, what specific transporters are required, and more specifically, how do ACNs exert their intracellular bioactivities. In our current CLSM and HPLC analyses with primary HUVECs and isolated thoracic aortas, the presence of Dp in the cellular plasma and vascular endothelium of mice after intravenous administration showed that the VECs absorbed Dp. Interestingly, SGLT1 was apparently expressed in the VECs and the active transport of Dp by HUVECs was dependent on SGLT1 activity. SGLT1 belongs to the Na (+)-glucose cotransporter (SGLT) family, which is widely expressed in human cell membranes, including small intestinal enterocytes and cardiac myocytes, and is responsible for sugar transport into cells. Reportedly, SGLT1 is necessary for the incorporation of the phenonic compound trans-piceid into human intestinal Caco-2 cells [54], [55]. Further, our present results suggest that SGLT1 is an important active transporter of Dp into VECs. Based on the above data and taking into account the volume of culture dishes (about 2 mL), Dp may attain an intracellular concentration higher than the initial extracellular concentration and the vascular endothelium may extend for up to about 240 m^2^, making it likely the largest tissue target of Dp [56], [36]. Furthermore, the Dp content in isolated thoracic aortas in our experimental model was well above the circulating levels post-administration, suggesting a previous indifference to Dp accumulation in some target tissues, in particular the blood vessels. Therefore, our results might explain the paradox that ACNs have a low bioavailability in humans but exert diverse bioactivities *in vivo*.

Another meaningful finding is that blockage of SGLT1 activity attenuated the protective effects of Dp on mitochondrial dysfunction. It is well known that mitochondria participate in the execution of apoptosis and also play an essential role in death signal transduction by the mPTP and collapse of the ΔΨm, resulting in the rapid release of Cyt c into the cytoplasm before binding to Apaf-1 and activating Caspase-3 via Caspase-9, thus culminating in cell death [57], [58] and AIF release from the mitochondria into the nuclei to continue the apoptotic pathway [59]. Other reports have shown that some Bcl-2 family members (such as Bax, Bcl-XL, Mcl-1, Bcl-2, and Bid) that are located on the mitochondrial membrane can alter the mPTP, which leads to caspase activation and subsequent apoptotic cell death [60]. In this study, Dp pretreatment significantly inhibited the release of AIF and Cyt c from the mitochondrial matrix into the cytosol but abolished by SGLT1 blockage or inhibition. And our results demonstrated a correlation between Dp and the preservation of mitochondrial function and further revealed the role of SGLT1 in Dp function in inhibition of mitochondrial dysfunction. Although the underlying mechanisms require further investigations, the present study demonstrated that Dp inhibited the intrinsic apoptotic signaling pathways.

In conclusion, these data offer new insights to further elucidate the biochemical mechanisms that underlie the reported impacts of dietary ACNs, particularly Dp, on the cardiovascular system. Nonetheless, further work is necessary to elucidate the protective efficacy of Dp in vivo and to clarify the molecular mechanisms involved.
