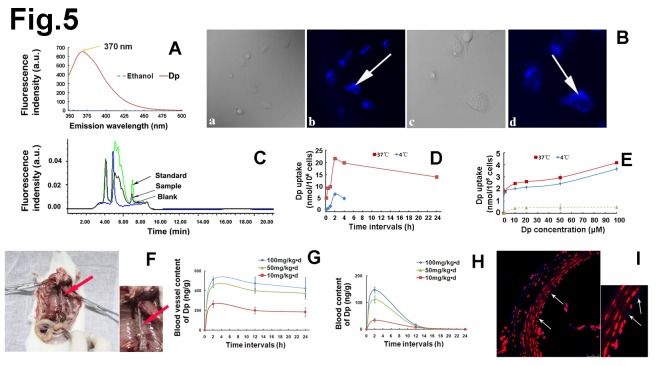# Correction: Delphinidin-3-Glucoside Protects against Oxidized Low-Density Lipoprotein-Induced Mitochondrial Dysfunction in Vascular Endothelial Cells via the Sodium-Dependent Glucose Transporter SGLT1

**DOI:** 10.1371/annotation/774b483b-cba3-46ce-a922-09d7f05487b0

**Published:** 2014-01-31

**Authors:** Xin Jin, Long Yi, Ming-liang Chen, Chun-ye Chen, Hui Chang, Ting Zhang, Li Wang, Jun-dong Zhu, Qian-yong Zhang, Man-tian Mi

An error occurred in Figure 5. The Y-axis title of Figure 5G should read "Blood vessel content of Dp (ng/g)" instead of "Blood content of Dp (ng/g)" and the Y-axis title of Figure 5H should read "Blood content of Dp (ng/L)" instead of "Blood vessel content of Dp (ng/g)."

Please see the corrected version of Figure 5 here: 

**Figure:**